# Bridging Inflammation and Neurodegeneration in Multiple Sclerosis: Mechanisms and Emerging Therapies

**DOI:** 10.7759/cureus.100941

**Published:** 2026-01-06

**Authors:** Aliasgar Taha

**Affiliations:** 1 Internal Medicine, Dr. Sulaiman Al Habib Hospital, Dubai, ARE

**Keywords:** btk inhibitors, compartmentalized inflammation, mitochondrial dysfunction, multiple sclerosis, neurodegeneration, progressive ms, remyelination

## Abstract

Multiple sclerosis (MS) is characterized by a complex interplay between inflammation and neurodegeneration that evolves over time. Although current immunotherapies effectively reduce relapses in relapsing-remitting MS, they fail to prevent long-term progression, particularly in progressive subtypes of MS. This review explores the mechanisms linking inflammation to axonal loss, with an emphasis on mitochondrial dysfunction, oxidative stress, and microglial activity. It also critically evaluates the limitations of existing disease-modifying therapies in addressing progression independent of relapse activity. Emerging central nervous system-penetrant strategies, including Bruton’s tyrosine kinase inhibitors, remyelinating agents, and neuroprotective compounds, are discussed as promising approaches to target compartmentalized pathology. Emphasis is placed on the need for integrated therapeutic approaches that target both inflammatory and degenerative disease processes. Finally, this review highlights the key knowledge gaps, advances in biomarker development, and future research directions that could guide the development of the next generation of MS therapies.

## Introduction and background

Multiple sclerosis (MS) is a chronic, immune-mediated neurodegenerative disease and a leading cause of neurological disability in young adults. The disease is characterized by autoimmune-mediated injury to the central nervous system (CNS), resulting in demyelination, axonal damage, and progressive neurodegeneration. MS most commonly presents as relapsing-remitting multiple sclerosis (RRMS), in which episodes of neurological dysfunction arise from inflammatory demyelinating lesions driven by the infiltration of T cells, B cells, and macrophages into the CNS. Although the precise initiating events in MS remain incompletely understood, inflammatory activity is thought to arise from a combination of genetic susceptibility and environmental triggers. Proposed mechanisms include immune responses to infectious agents, particularly viral infections, as well as molecular mimicry leading to autoimmunity against host antigens that cross-react with foreign antigens. These processes promote peripheral immune activation and subsequent CNS-directed inflammation, setting the stage for downstream neurodegeneration [1]. Over time, approximately 60-70% of patients with RRMS progress to secondary progressive multiple sclerosis (SPMS), where neurodegenerative processes and compartmentalized inflammation behind an intact blood-brain barrier (BBB) drive disease progression. Primary progressive multiple sclerosis (PPMS), which affects 10-15% of patients at onset, is defined by gradual disability accumulation from disease onset, with fewer overt inflammatory episodes but substantial cortical and axonal degeneration [1]. The transition from an inflammation-driven to a neurodegeneration-driven process underscores the complex and evolving pathophysiology of MS.

## Review

Methodology

This article is a narrative, mechanism-focused review that synthesizes key advances in the understanding of inflammation-neurodegeneration interactions in MS, rather than a systematic review conducted according to Preferred Reporting Items for Systematic reviews and Meta-Analyses (PRISMA) guidelines. This narrative review was developed through a targeted and iterative appraisal of the contemporary literature on MS, with emphasis on studies elucidating mechanisms linking inflammation to neurodegeneration and on emerging therapeutic strategies with translational relevance. Literature was identified through searches of PubMed, Scopus, and Web of Science, prioritizing peer-reviewed articles published in English within the past two decades, alongside seminal earlier studies where necessary for context. Given the conceptual and hypothesis-generating nature of this review, formal inclusion/exclusion criteria, risk-of-bias assessment, and PRISMA-guided study selection procedures were not applied. As this review is based on a narrative synthesis of heterogeneous mechanistic, preclinical, and clinical studies, no quantitative statistical analyses, meta-analyses, or pooled effect estimates were performed.

To frame subsequent mechanistic and therapeutic discussions, this section outlines the dual inflammatory and neurodegenerative processes that jointly drive disease progression in MS. MS is increasingly recognized as a disorder of dual pathology, wherein inflammatory and neurodegenerative processes interact dynamically to drive disease progression. In the early relapsing-remitting stage (RRMS), inflammation predominates, resulting in episodic demyelination and lesion formation. Over time, particularly as patients transition to SPMS or present with PPMS, neurodegeneration emerges as the principal driver of sustained disability. This process is fuelled by persistent and compartmentalized inflammation within the CNS [2-7].

In progressive MS, compartmentalized inflammation, referring to immune activity that becomes sequestered within the CNS, is characterized by the formation of tertiary lymphoid structures (TLS) in the meninges, consisting of B cells, T cells, and myeloid cells [4-7]. These structures are linked to cortical demyelination, gray matter atrophy, and accelerated disease progression [3,4,7]. Cytokines such as tumor necrosis factor-alpha (TNF-α), interleukin-17A (IL-17A), and interferon-gamma (IFN-γ), secreted by infiltrating lymphocytes and reactive glial cells, further exacerbate neuronal damage through activation of nuclear factor kappa B (NF-κB) and oxidative stress pathways [8-10]. Mitochondrial dysfunction plays a central role in amplifying this pathological cycle of injury [11-13]. Mitochondrial-derived damage-associated molecular patterns (DAMPs) released from injured axons perpetuate microglial activation and neuroinflammation [12]. In parallel, impaired nuclear factor erythroid 2-related factor 2 (Nrf2) signalling reduces the clearance of reactive oxygen species (ROS), thereby promoting sustained oxidative injury [8,9].

Astrocytes also contribute to this harmful response through mechanisms independent of direct cell-to-cell contact [10]. Reactive astrocytes release pro-inflammatory cytokines and disrupt synaptic homeostasis, thereby promoting neurodegeneration [10]. Even after treatments such as autologous hematopoietic stem cell transplantation, astrocyte markers such as glial fibrillary acidic protein (GFAP) stay elevated, showing that astrocyte activation is a long-lasting process [14,15].

While astrocytic activation and mitochondrial dysfunction illustrate how inflammatory and neurodegenerative mechanisms perpetuate injury at the cellular level, their impact is most apparent in the clinical consequences of dual pathology [7]. MRI studies demonstrate that cortical atrophy and gray matter volume loss are highly prevalent in progressive MS [5,6,16,17]. Diffusion and quantitative MRI further reveal pronounced microstructural changes that correlate with disability and cognitive decline [6,16,17]. Percentage brain volume change, a key clinical trial outcome, captures both white and gray matter atrophy and has emerged as a robust predictor of disease progression [16,17]. Importantly, progression independent of relapse activity (PIRA) is recognized as a distinct clinical manifestation of this dual pathology [6,7,18]. PIRA is observed early in the disease course and becomes the dominant driver of disability in progressive MS [6,7,18]. Predictors of PIRA include age, pre-existing disability, cortical atrophy, and infratentorial lesion burden [6,7,16,18].

Understanding the complex crosstalk between inflammation and neurodegeneration is critical to the development of effective therapies in MS [2,7,19]. Targeting cytokine activity, mitochondrial dysfunction, and glial activation may help shift the disease course and reduce disability progression [8-10,20-22].

Thus, the bidirectional interaction between inflammation and neurodegeneration not only drives clinical progression but also underpins the pathophysiological transition observed in progressive MS, where inflammatory activity gives way to neurodegeneration as the dominant mechanism.

Pathophysiology of progressive multiple sclerosis: from inflammation to neurodegeneration

Transition to a Neurodegenerative Disease State

The pathogenesis of MS involves a dynamic interplay between inflammation and neurodegeneration, which drives both relapsing and progressive disease forms [2,7,19]. Inflammation dominates the early relapsing-remitting phase (RRMS), whereas neurodegeneration and compartmentalized inflammation behind an intact BBB emerge as the dominant processes in the progressive phases of MS, encompassing SPMS and primary PPMS [2-7]. This transition represents a fundamental shift in pathology, from immune-mediated demyelination to neuronal and axonal injury driven by mitochondrial dysfunction, oxidative stress, and microglial activation [2,8,9,11,13]. Clarifying the mechanistic links between these processes is critical for developing therapies that can alter the long-term trajectory of MS [2,7,23].

Mechanistic Links Between Inflammation and Axonal Loss

Axonal degeneration is a key driver of disability progression in MS and is strongly associated with both acute inflammatory activity and chronic neurodegeneration [2,7,13]. Mitochondrial dysfunction and oxidative stress represent central mechanisms of axonal loss, generating a self-perpetuating cycle of energy failure, oxidative damage, and neuronal injury [8,9,11,13].

Mitochondrial Dysfunction in Multiple Sclerosis

Mitochondria are essential for neuronal health, regulating ATP production, calcium homeostasis, and oxidative balance. In MS, mitochondrial dysfunction contributes to axonal degeneration through several key mechanisms. Mitochondrial respiratory chain deficiencies, particularly in demyelinated axons, lead to compromised ATP production and impaired bioenergetics in MS [11,13,24]. This energy deficit disrupts axonal transport and accelerates axonal degeneration [13,24]. Respiratory chain impairment elevates ROS production, further contributing to neuronal injury [13,24,25]. Mitochondrial DNA (mtDNA) mutations and deletions identified in MS lesions impair respiratory chain function and promote neurodegeneration [26]. Mitochondrial-derived DAMPs exacerbate neuroinflammation and maintain microglial activation [12]. Abnormal mitochondrial transport and distribution within demyelinated axons impair energy delivery to axonal segments, thereby exacerbating degeneration [13,24]. Moreover, mitochondrial calcium (Ca²⁺) signalling is essential for maintaining bioenergetic balance [11]. Disrupted Ca²⁺ homeostasis via impaired endoplasmic reticulum-mitochondria-associated membranes contributes to mitochondrial dysfunction, oxidative stress, and axonal loss [11,13]. Novel approaches, such as transferring healthy mitochondria into cells and employing mitochondria-targeted antioxidants, show potential for reducing mitochondrial damage and protecting axons [8,9,27]. Closely linked to mitochondrial dysfunction, oxidative stress plays a pivotal role in amplifying neuronal injury and sustaining neuroinflammation in MS.

Oxidative Stress and Neuroinflammation

Oxidative stress, driven by excessive ROS and reactive nitrogen species (RNS), represents a major contributor to neuronal and axonal injury in MS [8,11,26]. ROS generated by dysfunctional mitochondria initiate lipid peroxidation, resulting in the formation of toxic aldehydes, such as 4-hydroxynonenal [11,26]. These compounds damage axonal membranes, impair synaptic function, and trigger axonal loss [13,24,26]. Oxidative stress also induces protein misfolding and aggregation, thereby disrupting cellular homeostasis and promoting neurodegeneration [26]. Lipid peroxidation products modulate pathways, including apoptosis, autophagy, and ferroptosis, thereby exacerbating axonal injury [8,26]. Biomarkers such as neurofilament light chain (NfL) reflect oxidative damage and correlate with clinical disability [28-32]. Antioxidant-based therapies, peroxisome-targeted interventions, and ferroptosis inhibitors are emerging as potential strategies to counteract oxidative injury and preserve axonal health [8,9,33]. The interplay between mitochondrial dysfunction, oxidative stress, and axonal injury is summarized in Table 1.

**Table 1:** Mechanistic links between inflammation and neurodegeneration in MS. Summary of key pathological features and mechanisms bridging inflammatory and neurodegenerative processes in MS. These include mitochondrial dysfunction, oxidative stress, and compartmentalized CNS inflammation. BBB = blood-brain barrier; CNS = central nervous system; DAMPs = damage-associated molecular patterns; MS = multiple sclerosis; ROS = reactive oxygen species; RNS = reactive nitrogen species; PIRA = progression independent of relapse activity; TLS = tertiary lymphoid structure

Mechanism	Key features	Evidence in MS pathology	Relevance to progression
Mitochondrial dysfunction	Energy failure, ROS generation, calcium overload	Seen in demyelinated axons and chronic active lesions	High
Oxidative stress	ROS/RNS-mediated damage to lipids, proteins, and DNA	Elevated in chronic MS plaques	High
Microglial activation	Pro-inflammatory phenotype, complement activation	Prominent in gray matter and at lesion edges	High
Iron deposition	Catalyzes ROS via the Fenton reaction, promotes inflammation	Noted in the deep gray matter and at lesion rims	Moderate–high
Compartmentalized inflammation	Inflammation trapped behind an intact BBB, TLS in the meninges	Associated with cortical thinning and PIRA	High

Role of Microglia and Compartmentalized Inflammation

In progressive MS, inflammation is compartmentalized behind an intact BBB, sustaining a chronic neurodegenerative state resistant to current immunotherapies [2-4,7]. Activated microglia, the resident immune cells of the CNS, play a central role in this process [2,7]. Iron accumulation within microglia is recognized as a hallmark of MS lesions and promotes a sustained pro-inflammatory phenotype [34]. Iron-loaded microglia demonstrate impaired mitochondrial function, increased ROS production, and heightened neurotoxicity [34]. Ferroptosis within microglia further amplifies neurodegeneration [8,34]. Moreover, microglia in progressive MS upregulate major histocompatibility complex class II and interferon type I pathways, contributing to neuronal injury [2,7,35]. Deficient production of specialized pro-resolving lipid mediators perpetuates microglial activation and chronic inflammation [23]. Therapeutic strategies aimed at targeting iron metabolism and modulating microglial phenotypes may provide new avenues for intervention in progressive MS [2,34,36].

Gray Matter Demyelination

**Figure 1:**
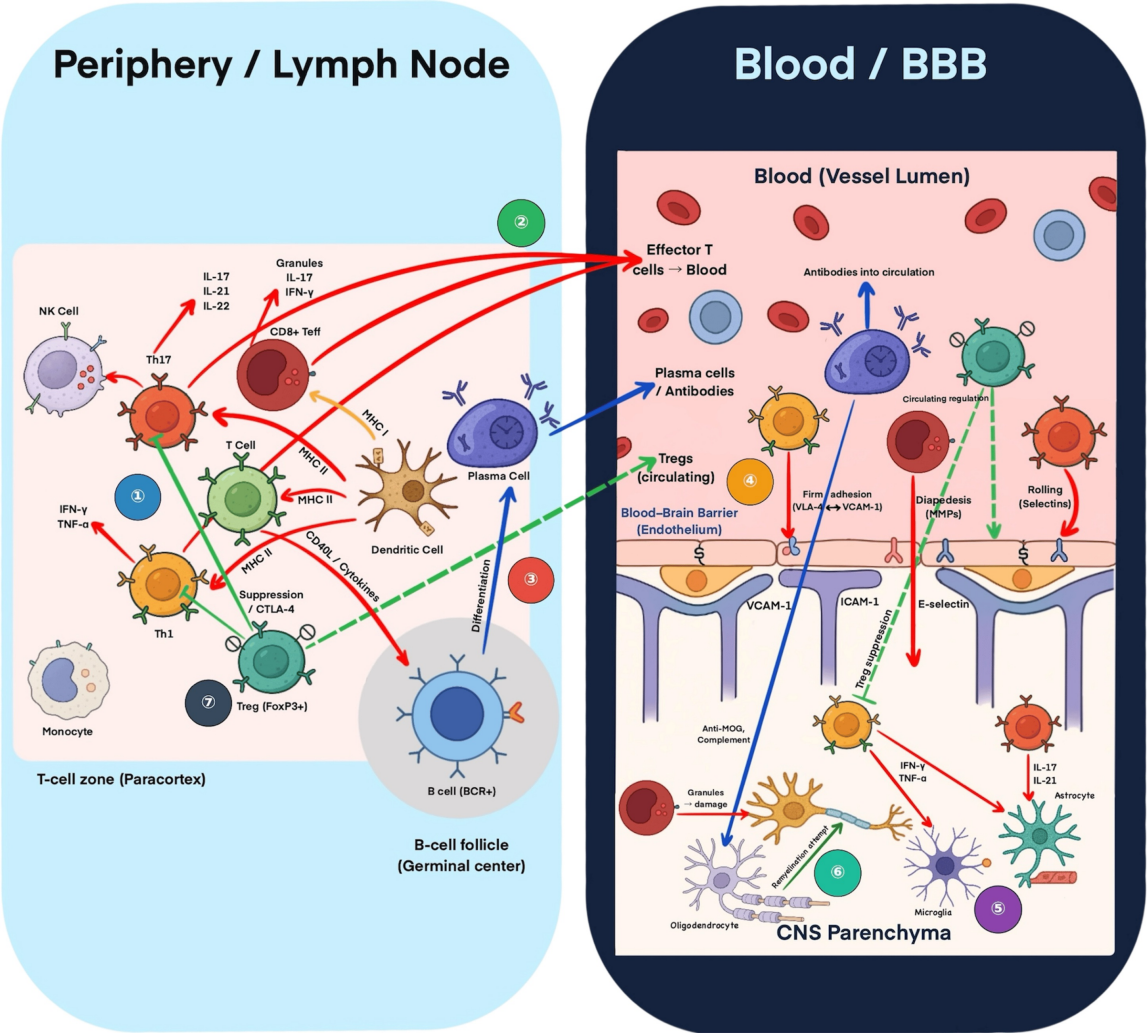
Immunopathogenesis and therapeutic strategies in MS, illustrating the continuum from peripheral immune activation to CNS infiltration and tissue injury. This schematic illustrates immune events driving MS across the periphery, BBB, and CNS. In lymph nodes, naïve T cells differentiate into Th1, Th17, and CD8⁺ effector T cells, while B cells develop into plasma cells. Regulatory Tregs provide suppression but may be functionally impaired. Activated lymphocytes enter the bloodstream and interact with adhesion molecules (VLA-4/VCAM-1, ICAM-1, E-selectin) to cross the BBB via rolling, adhesion, and diapedesis. In the CNS, infiltrating lymphocytes, antibodies, complement, microglia, and astrocytes promote demyelination and neurodegeneration through cytokines such as IFN-γ, TNF-α, and IL-17. Therapeutic classes are shown numerically: (1) agents reducing T-cell activation; (2) S1P modulators; (3) anti-CD20 therapies; (4) natalizumab; (5) BTK inhibitors; (6) remyelination/neuroprotective strategies; (7) cell-based tolerance therapies. BBB = blood–brain barrier; BCR = B-cell receptor; BTK = Bruton’s tyrosine kinase; CNS = central nervous system; CTLA-4 = cytotoxic T-lymphocyte–associated antigen 4; DC = dendritic cell; ICAM-1 = intercellular adhesion molecule-1; IFN-β = interferon-beta; IFN-γ = interferon-gamma; IL = interleukin; MHC I/II = major histocompatibility complex class I/II; MOG = myelin oligodendrocyte glycoprotein; MS = multiple sclerosis; MSC = mesenchymal stem cell; NK cell = natural killer cell; S1P = sphingosine-1-phosphate; Treg = regulatory T cell; TNF-α = tumor necrosis factor-alpha; VCAM-1 = vascular cell adhesion molecule-1; VLA-4 = very late antigen-4. This figure is original and created by the author; no published material was reproduced or adapted.

Gray matter pathology is a key feature of progressive MS and is strongly linked to meningeal inflammation [4,6,7]. Pro-inflammatory cytokines, such as IFN-γ, TNF-α, and IL-17A, promote cortical gray matter demyelination through direct neuronal effects, astrocyte activation, and complement pathway engagement [3,35]. Activated astrocytes further disrupt synaptic homeostasis and compromise BBB integrity [10,19]. Complement components are detected in cortical lesions and contribute to ongoing demyelination [3,37]. Clinically, MRI studies have shown that cortical lesions and gray matter atrophy correlate with cognitive impairment and disability progression in MS [16,17]. Diffusion MRI and quantitative susceptibility mapping reveal microstructural gray matter changes that are closely associated with cognitive trajectories [17,19]. A deeper understanding of the molecular drivers of gray matter pathology could guide strategies to promote remyelination and preserve cognitive function in patients with progressive MS [5,7]. A schematic overview linking these immunopathogenic mechanisms to current and emerging therapeutic targets is shown in Figure 1.

Limitations of current immunotherapies and the need for central nervous system-penetrant strategies

Limitations of Current Disease-Modifying Therapies

Despite the introduction of more potent disease-modifying therapies (DMTs) over the past two decades, current treatments have had little impact on the transition from RRMS to SPMS [6,38]. Most DMTs, including monoclonal antibodies (MAbs) and sphingosine-1-phosphate (S1P) receptor modulators, penetrate the CNS poorly and cannot target compartmentalized inflammation or promote neuroprotection [39-42]. Consequently, although DMTs reduce relapse rates and new lesion formation in RRMS, they do not prevent long-term disability progression in progressive forms of MS [18,38,43-46]. These limitations have prompted increasing efforts to develop CNS-penetrant therapies that can address both inflammation and neurodegeneration [35,40,42].

Pharmacokinetic and Molecular Barriers to Central Nervous System Penetration

DMTs primarily act on peripheral immune mechanisms, restricting their capacity to address chronic inflammation and neurodegeneration within the CNS [6,7,38]. Pharmacokinetic and molecular barriers imposed by the BBB limit the CNS availability of these agents [39,40,42]. Accordingly, most DMTs, including MAbs and S1P receptor modulators, are designed to act on peripheral immune cells [41,43-45]. Ocrelizumab targets CD20-positive B cells, reducing inflammatory lesion activity and relapse rates, but demonstrates limited efficacy in preventing cortical thinning and axonal degeneration in progressive MS [38,43,46,47]. Natalizumab blocks α4-integrin to prevent leukocyte migration across the BBB, reducing relapse frequency but not altering the trajectory of gray matter atrophy or synaptic loss [38,44,48,49]. Fingolimod sequesters lymphocytes in lymph nodes via S1P receptor modulation, reducing relapse rates but exerting limited effects on progressive neurodegeneration [38,41,45,50,51]. Natalizumab is detectable in the cerebrospinal fluid (CSF) at low concentrations (1.5 ng/mL), though the clinical significance of this penetration remains uncertain [48,52]. Ocrelizumab and fingolimod exert limited or indirect CNS effects, showing poor CSF penetration relative to plasma levels [40-42,46]. These findings highlight that peripheral immune modulation alone is insufficient to halt progression; CNS-compartmentalized inflammation persists behind an intact BBB, driving gray matter pathology and disability [2-4,7].

Innovative Strategies to Overcome Blood-Brain Barrier Limitations

The BBB is a highly selective barrier that restricts the passage of large hydrophilic molecules, such as MAbs and small-molecule modulators, into the CNS [39,40]. At ~150 kDa, MAbs are too large for effective passive diffusion across the BBB [39]. Receptor-mediated transcytosis has been explored as a means to enhance CNS delivery, but its clinical translation remains limited [40,42]. In addition, efflux transporters, such as P-glycoprotein and multidrug resistance-associated proteins, are abundantly expressed at the BBB, actively exporting drugs back into the bloodstream [40]. CSF biomarker studies show that although ocrelizumab reduces serum neurofilament light chain (sNfL) and peripheral inflammation, residual CSF markers of compartmentalized inflammation (e.g., GFAP, CHI3L1, CXCL13) persist during treatment [14,15,28-30,53-57]. Natalizumab demonstrates similar limitations, as disease reactivation is frequently observed upon cessation despite initial peripheral immune suppression [44,48,52]. Thus, pharmacokinetic barriers and persistent CNS inflammation account for the inability of current DMTs to halt disability progression, particularly in SPMS and PPMS [6,7,38].

Several strategies are being investigated to enhance CNS drug delivery [42]. Lipid-based and polymeric nanoparticles are being developed to improve the CNS penetration of DMTs by increasing drug stability and circumventing efflux transporters [42]. Engineering MAbs to target transferrin or insulin receptors represents a promising approach to enable receptor-mediated transcytosis across the BBB [40,42]. Direct CNS delivery, including intrathecal administration of MAbs or small molecules, is under investigation to bypass the BBB and increase local CNS drug concentrations [42].

Progression Independent of Relapse Activity

PIRA is increasingly recognized as a distinct phase of MS, driven by neurodegeneration and compartmentalized inflammation, rather than acute inflammatory relapses [7,18]. Current DMTs show limited efficacy in preventing PIRA, highlighting their inability to adequately target the underlying drivers of progression [6,38]. Ocrelizumab reduces upper extremity impairment progression and lowers sNfL levels, yet CSF biomarkers indicate incomplete control of compartmentalized inflammation [30,46,47,55]. Natalizumab sustains low relapse rates but does not halt cortical atrophy or gray matter demyelination, and CSF biomarkers similarly indicate residual inflammation [17,44,48,55]. Fingolimod reduces relapse rates in RRMS but lacks efficacy against long-term disability progression and does not meaningfully modulate CNS-compartmentalized inflammation [41,42,45,51].

Bridging to Emerging Central Nervous System-Penetrant Immunotherapies

Bruton’s tyrosine kinase (BTK) inhibitors represent a promising class of CNS-penetrant small molecules that may overcome the limitations of current DMTs [20-22]. BTK inhibitors such as tolebrutinib and evobrutinib demonstrate measurable CSF penetration and modulate CNS-resident immune cells, including microglia and B cells [21,58,59]. In early phase trials, tolebrutinib (60 mg/day) and evobrutinib (75 mg twice daily) demonstrated favorable CNS bioavailability and sustained BTK occupancy [58,59]. Ongoing phase III trials are expected to determine whether BTK inhibitors can alter the course of progressive MS by targeting compartmentalized inflammation [21,38]. These emerging CNS-penetrant strategies provide a pathway to address the dual pathology of MS and remain a central focus for future therapeutic development [21,22].

Together, these developments underscore a paradigm shift: effective management of progressive MS will depend not only on immunotherapies that penetrate the CNS but also on novel strategies that simultaneously address inflammation and neurodegeneration.

Emerging therapeutic strategies targeting both inflammation and neurodegeneration

Given the breadth of neuroinflammatory and neurodegenerative mechanisms implicated in MS, this review does not attempt to provide an exhaustive recapitulation of all molecular pathways. Instead, it adopts a focused, mechanistically driven approach, using CNS-penetrant BTK inhibition as a paradigmatic example of how targeted immunomodulation may simultaneously influence compartmentalized inflammation and downstream neurodegeneration. BTK inhibitors were selected because they uniquely bridge peripheral B-cell biology and CNS-resident microglial activation, two processes increasingly recognized as central to progressive MS pathology.

An expanding understanding of MS as a dual pathology driven by both inflammation and neurodegeneration has prompted the development of therapeutic strategies that seek to modulate immune responses while protecting the neural tissue. Current DMTs primarily target peripheral immune mechanisms but are of limited efficacy against CNS-compartmentalized inflammation and neurodegeneration. Accordingly, a new generation of therapies is emerging, including CNS-penetrant immunomodulators, neuroprotective agents, remyelination-enhancing compounds, and combination approaches designed to address the multifaceted pathophysiology of MS.

Bruton’s Tyrosine Kinase Inhibitors and Central Nervous System-Penetrant Immunotherapies

BTK inhibitors are among the most promising classes of CNS-penetrant immunomodulatory agents for MS [20,21]. This class was selected as a focal point in the present review because BTK sits at the intersection of peripheral B-cell activation and CNS-resident microglial signalling, thereby providing a mechanistically coherent framework to examine how targeted immunomodulation may influence both neuroinflammation and downstream neurodegeneration. BTK is expressed in both B cells and CNS-resident microglia, which drive compartmentalized inflammation and neurodegeneration in progressive MS [20,21]. Several BTK inhibitors, including evobrutinib and tolebrutinib, can cross the BBB and modulate microglial activity [21,58,59]. BTK inhibitors may reduce neuroinflammation and promote neurorepair by shifting microglia from a pro-inflammatory (M1) to an anti-inflammatory (M2) phenotype [20,21]. BTK inhibition also dampens B-cell antigen presentation and cytokine production within the CNS, potentially mitigating TLS formation and gray matter demyelination [20,21]. Phase II trials of evobrutinib and tolebrutinib have shown significant reductions in new and enlarging MRI lesions, with tolebrutinib showing durable clinical efficacy over two years [58,59]. Ongoing Phase III trials are assessing whether BTK inhibitors can alter the trajectory of progressive MS by targeting CNS-compartmentalized inflammation [21,59]. Preliminary safety data suggest that BTK inhibitors are well tolerated, employing non-depleting mechanisms that preserve peripheral immune surveillance while modulating CNS immune activity [20,21,58,59].

Neuroprotective and Remyelinating Strategies

Given the critical role of neurodegeneration in MS progression, there is an urgent need for therapies that protect neurons, support mitochondrial function, and promote remyelination [7,8,13]. Mitochondrial dysfunction is a key driver of axonal degeneration in MS [11,13,24]. Novel therapeutic approaches seek to restore mitochondrial integrity and function [25,27,33]. Mitochondria-penetrating peptides and nanoparticle-based delivery systems are under development to enhance mitochondrial drug delivery, though challenges in specificity and long-term safety persist [39,40,42]. Interpatient variability in mitochondrial dysfunction highlights the need for personalized mitochondrial-targeted therapies [7,60,61]. Beyond mitochondria, emerging strategies focus on enhancing synaptic plasticity and promoting remyelination [62-67]. Mesenchymal stem cell therapies promote neuroprotection and remyelination, with early phase clinical trials demonstrating improvements in Expanded Disability Status Scale scores and biomarkers of neural repair [57]. Small molecules, such as digoxin and ursolic acid, have been shown to promote oligodendrocyte differentiation and myelin repair in preclinical models [63,65]. Nanotechnology-based therapies, including gold nanocrystals and biomaterial-based vaccines, have the potential to enhance oligodendrocyte maturation and induce immune tolerance [42]. Synaptic repair is also being investigated through neural precursor cell (NPC) transplantation, which has been associated with reduced brain atrophy and enhanced neuroprotective biomarker profiles in patients with progressive MS [57].

Combination Therapies

Combination strategies that integrate immunomodulation with neuroprotection represent a promising avenue for MS treatment [22,27,62,68]. NPC transplantation exerts both neuroprotective and immunomodulatory effects, as evidenced by increased anti-inflammatory and neuroprotective CSF molecules in clinical trials [57]. TNF receptor modulation, specifically TNFR1 blockade combined with TNFR2 stimulation, enhances neuroprotective signalling while reducing demyelination and inflammation in preclinical models [69]. Glatiramer acetate demonstrates both immunomodulatory and neuroprotective effects, mitigating cognitive decline and promoting neural tissue resilience [70]. Vitamin D supplementation provides neuroprotective and immunomodulatory benefits, promoting remyelination and preserving BBB integrity [71]. Dual inhibitors (e.g., GSK3β/PDE7) and S1P receptor modulators (e.g., ozanimod) provide complementary immunological and neuroprotective effects, underscoring their potential in combination regimens [41,62,72]. Despite these advances, significant challenges to effective implementation remain [38]. The MS-SMART trial underscored the difficulty of achieving neuroprotection with monotherapies, indicating that future progress will likely require rationally designed combination therapies targeting multiple disease mechanisms simultaneously [68].

Table 2 summarizes the key therapeutic strategies targeting both inflammation and neurodegeneration in MS.

**Table 2:** Emerging therapeutic strategies targeting dual pathology in MS. Overview of CNS-penetrant and neuroprotective therapies under investigation for MS, including their mechanisms of action and current trial status. aHSCT = autologous hematopoietic stem cell transplantation; BTK = Bruton’s tyrosine kinase; CNS = central nervous system; EAE = experimental autoimmune encephalomyelitis; ER = endoplasmic reticulum; GFAP = glial fibrillary acidic protein; MS = multiple sclerosis; NfL = neurofilament light chain; PDE = phosphodiesterase

Therapeutic strategy	Primary targets	Stage of development	Mechanism of action	Potential impact
BTK inhibitors	B cells, microglia	Phase II/III	Inhibit BCR signaling and CNS-compartmentalized inflammation	High
Clemastine	Oligodendrocyte precursor cells	Phase II	Promotes remyelination in CNS	Moderate
VP3.15	Mitochondria, PDE7/GSK3β	Preclinical	Improves mitochondrial bioenergetics, reduces inflammation	Moderate–high
Combination therapy (e.g., IFN-β + PCB)	Cytokines, remyelination	Preclinical (EAE)	Reduces IL-6/IL-17, promotes myelin repair	High
aHSCT	Broad immune reset	Clinical	Restores immune tolerance, reduces NfL, GFAP	High

While emerging immunomodulatory, neuroprotective, and combination strategies highlight the therapeutic potential of targeting both arms of MS pathology, several critical questions remain unanswered. These limitations underscore the need to explore future directions and address key research gaps.

Future directions and research gaps

Despite significant advances in immunotherapy for MS, effective treatments that target neurodegeneration and promote repair remain a major unmet need, particularly in patients with progressive MS. Several key challenges must be overcome to advance therapeutic success in this area.

Oligodendrocyte Precursor Cell and Extracellular Matrix Barriers

A major barrier to progress is the complex, multifactorial nature of remyelination failure in progressive MS [5,7,38]. Intrinsic failures in oligodendrocyte precursor cell (OPC) differentiation play a central role in this process. Extracellular HMGB1 impairs OPC maturation via TLR2/4-NFκB signaling [73], while inhibitory proteins such as Nogo-A and LINGO-1 suppress oligodendrocyte differentiation [74]. The extracellular matrix within MS lesions adds further barriers, as fibronectin and versican-V1 create a non-permissive environment that hinders OPC migration and differentiation [75,76].

Microglia, miRNAs, and Bone Morphogenetic Protein Pathways

Microglial dysfunction further impairs repair. Deficient TREM2 signaling compromises myelin debris clearance and promotes chronic pro-inflammatory states, while disrupted microglia-OPC crosstalk further impedes functional remyelination [2,73,74]. Additional contributors include miRNA dysregulation (e.g., miR-223 and miR-124) and activation of the bone morphogenetic protein pathway, both of which block OPC differentiation and repair [75,76]. Targeting these mechanisms may help establish a permissive environment for repairs.

Another major limitation is that current preclinical models fail to fully capture the pathophysiology of progressive MS [38,62]. Most existing experimental autoimmune encephalomyelitis models emphasize acute inflammation and relapsing disease, but fail to reproduce the chronic neurodegeneration that characterizes progressive MS [27,63,64]. Conventional imaging approaches in animal models lack the resolution to detect subtle axonal and myelin injuries. Moreover, critical factors such as aging, microglial priming, and chronic inflammation remain underexplored [17,19]. Improved models that incorporate advanced imaging, validated biomarkers, and a stronger focus on microglial dynamics are critically needed to enhance translational relevance [14,77,78].

Designing effective clinical trials for neuroprotective therapies in progressive MS remains a major challenge [38]. The slow and variable course of neurodegeneration necessitates long-duration trials with highly sensitive outcome measures [38,68]. The multiarm MS-SMART trial highlighted the limitations of the current endpoints and underscored the need for greater mechanistic diversity in candidate therapies [68]. The EXPAND trial demonstrated the value of integrating imaging endpoints (gray matter atrophy, magnetization transfer ratio) and fluid biomarkers (NfL, CXCL13) to enhance sensitivity to treatment effects [53-55,79-81]. Early phase trials are critical for refining candidate therapies and identifying responsive patient subgroups. Recent studies on neural stem cells and agents such as EGCG exemplify this approach, although the results have been mixed [57,82]. Overall, future trials should adopt flexible, adaptive designs, incorporate multimodal biomarker panels, and stratify patients by biological and clinical markers to maximize the likelihood of detecting meaningful neuroprotective effects [38,77,78,83].

Biomarker development will be critical for advancing therapeutic strategies in MS [38]. Serum and CSF NfL is the most validated biomarker of axonal injury and treatment response, although its interpretation is complicated by confounding factors such as age and comorbidities [28-32]. Other emerging biomarkers include CHI3L2, which is associated with disability progression 55, and myelin basic protein, which reflects remyelination dynamics [84]. Complementary markers such as RTN4, tau, and GFAP capture additional aspects of neurodegeneration and astrocyte activation, but require further validation before clinical adoption [15,29,77,78]. Integrating these biomarkers into clinical trials and routine care will enable more precise monitoring of disease progression and therapeutic response [55,83,85].

Finally, advances in precision medicine offer new opportunities for individualized MS care [38,86]. NfL has already been employed to monitor treatment response and disease activity, though interpretation must be carefully contextualized [28-32]. Myeloid-derived suppressor cells, particularly the monocytic subset, are emerging predictors of treatment efficacy, especially in response to fingolimod [36,50]. Extracellular vesicle-derived miRNAs also provide insights into disease stage and pathophysiology and may support future patient stratification [84,85]. The successful implementation of biomarker-guided therapy in MS will require assay standardization, validated clinical cut-off points, and large-scale validation across diverse populations [78,81]. Ultimately, precision-guided neuroprotective and repair-promoting strategies represent a key frontier in MS therapy development [22,35,38].

## Conclusions

Progressive MS remains a major therapeutic challenge due to its complex and incompletely understood pathophysiology, characterized by neurodegeneration, compartmentalized innate immune activity, and a gradual, heterogeneous transition from relapsing disease. Current DMTs provide only limited benefit in this stage, in part because they were not designed to target progressive mechanisms and because patients with progressive MS have historically been underrepresented in clinical trials. Additional challenges include the dual and context-dependent roles of microglia and macrophages, the absence of validated biomarkers to detect early progression, and persistent translational gaps between experimental models and clinical outcomes. Collectively, these factors underscore the need for deeper mechanistic understanding and innovative trial designs tailored specifically to progressive MS. Emerging therapeutic strategies targeting both neuroinflammation and neurodegeneration offer cautious optimism. Approaches such as B-cell depletion, CNS-penetrant BTK inhibitors, microglial modulation, and neuroprotective agents aim to address the dual pathology of MS, while non-canonical targets may complement established immunotherapies. In parallel, precision medicine strategies integrating advanced imaging, fluid biomarkers, and machine-learning tools hold promise for improving disease stratification and guiding earlier, individualized intervention. Meaningful progress will likely require combinatorial treatment strategies and robust biomarker validation, alongside assessment of real-world, patient-centered outcomes. Aligning mechanistic advances with clinical applicability will be essential to improving long-term functional outcomes and quality of life for individuals with progressive MS.
